# The Prevalence of Dental Caries in Primary Dentition in 4- to 5-Year-Old Preschool Children in Northern Palestine

**DOI:** 10.1155/2014/839419

**Published:** 2014-09-23

**Authors:** Zafer Azizi

**Affiliations:** Pediatric Dentistry Department, Arab American University, Jenin, Palestine

## Abstract

*Aim.* To determine the prevalence of dental caries among a representative sample of preschool children (4-5 years old) who were accompanied by their parents to the dental centre of the Arab American University in Jenin whether they come seeking dental treatment or as visitors with adult patients. *Materials and Methods.* 1376 children of both sexes were investigated by three calibrated and trained examiners for dental caries using the dmft index according to the WHO method. *Results.* 76% of the studied children have already experienced dental caries at the age of 4-5 years (1046 children). The mean dmft score was found to be 2.46 while the other 24% of children were caries-free. There was no significant difference in caries prevalence between boys and girls (77.2% versus 74.6%). Children of highly educated and college graduated mothers were found to have more fillings (restored teeth) in comparison to those who belong to mothers who did not finish their secondary (high school) education. *Conclusion.* The number of caries-free children in northern Palestine is still far from numbers found in developed countries. There is a real need to make improvements at the level of parents dental health education, application of preventive measures, and dietary habits among preschool children.

## 1. Introduction

Dental caries in primary teeth has been widely studied in many countries worldwide since it is known to be one of the most common oral diseases of childhood [1]. However, figures to quantify the prevalence of such disease in Palestine are almost inexistent.

It is extremely important to study the prevalence of dental caries in preschool children in Palestine to be able to provide a clear picture about the situation of oral and dental health in such population so as to help the local authorities to make future plans to reduce as much as possible the incidence of dental caries.

Many countries have developed different strategies to reduce or even eradicate dental caries from preschool children following dental caries surveys. Some have already obtained excellent results using water and salt fluoridation, dental health education, school oral health program, and so forth.

In Palestine, dental caries surveys are rare and are unable to draw the attention of the local health authorities to take any reaction in fighting such common disease.

The aim of this study is to give an idea about the actual situation of dental caries among preschool children in Palestine. The results found here would probably be helpful in making future plans concerning the best methods to lower the level of such oral disease and make necessary improvements in the Palestinian health system especially in the field of oral and dental health problems.

The prevalence of dental caries in primary teeth is commonly evaluated using the dmft index. The number of decayed, missing, and filled primary teeth is calculated in each child to obtain a sum that is known to be the mean dmft score in such child [2].

## 2. Materials and Methods

Children of both sexes aged between 4 years and 5 years (*n* = 1376) who were accompanied by their parents to the dental centre of the Arab American University of Jenin during the first 10 months of 2013 were examined clinically by one of the three calibrated examiners in the clinics of Pediatric Dentistry Department after gaining the permission of the parents.

As the families come from different geographic areas of northern Palestine, the examined population was subdivided as shown in Table 1. Medical and dental histories were taken for each child by the help of one of the parents before starting the clinical examination. The parent was asked about the mother's level of education (elementary school, secondary school, college graduated, or postgraduate studies).

Children who had previous history of receiving any specific organized preventive treatment were excluded from the study.

Clinical examination intraorally was achieved using sterile dental mirror and sterile dental explorer under dental unit's light. The presence of cavitation has been considered to be indicative of carious lesion in accordance with the criteria recommended by the WHO in 1997 [2–4].

dmft index has been used to measure the prevalence of caries activity. Teeth which were missing due to trauma or congenitally absent teeth were excluded from the data processing; therefore, they did not contribute to the final score. Missing teeth were counted only when their loss was due to caries.

The number of dental restorations has also been registered for each child which contributed to the F component in the dmft score.

## 3. Results

The dmft scores of the clinically examined children were registered as shown in Table 2.

It was found that only 330 children (24%) were caries-free (dmft = 0) at the age of 4-5 years while the other 1046 children have already experienced dental caries at this age.

**Figure 1:**
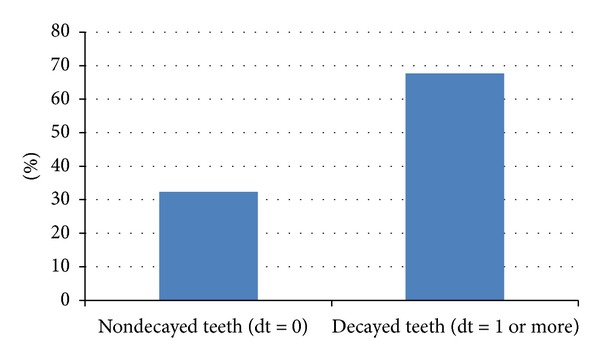
Prevalence of decayed teeth.

As for the (d) component, it was found that 932 children have had at least dental decay or caries in one or more primary teeth at the cavitation stage by the age of 4-5 years as shown in Figure 1.

**Figure 2:**
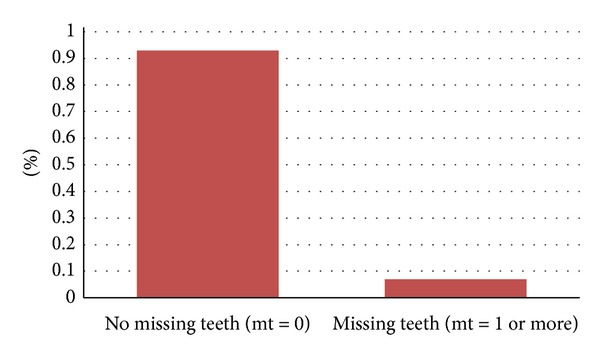
Prevalence of missing teeth.

However, the number of decayed teeth in the whole population studied here was 2895. The mean dt would be 2.10 while the number of children who had at least one missing primary tooth due to dental caries was only 98 representing 7.1% of the overall sample. The figure below shows the prevalence of missing teeth (mt) in the studied population (Figure 2).

The number of missing teeth in the whole studied population was 163. Thus, the mean mt is 0.12.

**Figure 3:**
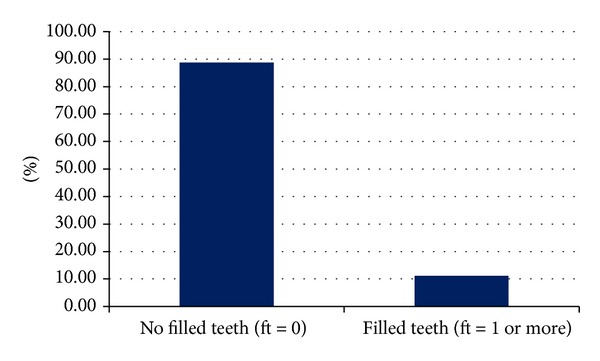
Prevalence of filled teeth.

When looking to the number of filled teeth representing the (f) component here, it was found that the number of children who had at least one restored primary tooth (filled tooth) was 154 representing 11.2% of the whole sample as shown in Figure 3, while the total number of restored teeth was 325.

This means that the mean ft is 0.24.

When asked about their level of education, mothers of examined children were divided into 4 groups as shown in Table 3.

As mentioned above, the number of children who had at least one restored (filled) tooth was 154. Mothers of such children have been found to have a high level of education in comparison to mothers of children who have decayed (cavitated) teeth or missing teeth (Table 4).

Correlation between mother's level of education and the presence of fillings in her child's mouth has been found to be statistically significant (*P* value < 0.05).

The overall caries prevalence in the study population was 76% with an overall mean dmft score of 2.46 of which decayed component is 2.10, missing component 0.12, and filled component 0.24.

Difference between males and females in caries prevalence at the age of 4-5 years has not been found to be significant as demonstrated by Table 5. As seen above, caries prevalence in primary teeth of 4- to 5-year-old girls is 74.6% which is very close to that of boys at the same age (77.2%).

The table below shows the mean dmft scores for both sexes (Table 6).

## 4. Discussion

The prevalence of dental caries in primary dentition in 4- to 5-year-old children in northern Palestine would be about 76% which means that almost 3 children out of 4 have already experienced dental caries by the age of 5 years in northern Palestine.

Figures found here seem to be far from the WHO/FDI goals for 2000; that is, 50% of 5- to 6-year-old children should be caries-free [5].

When compared to other developing countries, recent studies in Pakistan and India revealed that caries prevalence in preschool children in different regions of both countries is about 50–60% which is considered much better than what we found in our study here [6–9].

On the other hand, caries prevalence in preschool children in some Arab countries like Saudi Arabia has been found to be high (approaching the 75%) [10–12].

In the United Arab Emirates, a high prevalence of caries among preschool children has been registered as well (70–80%) [13, 14]. Kuwaiti kindergarten schoolchildren who are caries-free at the age of 4-5 years do not represent more than 24–32% of such population according to a national epidemiologic survey done in Kuwait in 2010 [15]. These findings are almost similar to what we found here in northern Palestine, although they are still far from the figures published by many developed countries as we could find in the United Kingdom (40–60% caries prevalence in 5-year-old children) or in Sweden (69% of 3-year-old preschool children are caries-free in 2003) as well as in Brisbane, Australia (66% of 4- to 6-year-old children are caries-free in 2002) [16–18].

A probable explanation for such discrepancy can be the following: inequality in economic conditions and resources, effective fluoridation policy, efficiency of healthcare system, availability and consumption of refined sugars, standard of oral health awareness among public, dietary and oral hygiene lifestyles, and motivation status of parents and children [6].

As expected, highly educated mothers tend to take their children to the dentist early in their lives so as to make regular check-ups and treat dental caries as soon as it appears, which best explains the high f component (ft) in case of children of college-graduated mothers [19].

## 5. Conclusion

The mean dmft scores as well as caries prevalence in primary teeth of 4- to 5-year-old Palestinian children reflect a considerable defect in the oral health care at home and at school, showing a real need to establish “School Oral Health Programs” in the different regions of northern Palestine [1, 7].

Such programs are possible to implement in cooperation with the Ministry of Health so as to be able to finance them properly [15].

It is always possible to prevent dental caries in primary dentition and lower the caries prevalence in young children in northern Palestine starting with good dental health education of the parents and teaching them how to take care of their children's teeth as soon as they start to erupt. Emphasis on babies feeding habits as well as the use of kids toothpastes is also important [1, 20].

Parents should be encouraged to take their children to the public dental clinics directed by the ministry of health or to their private dentist before the age of 1 year.

They would be able to have an idea about dental health education programs and topical fluoride application campaigns.

Mobile dental clinics would probably help in reaching rural areas that lack dental services.

Preventive measures campaigns including topical fluoride application, fissure sealants, and healthy diet promotion would be a lot of help to improve the situation of the oral and dental health in young children who go to nurseries and kindergartens.

It is always possible to find volunteer dentists locally or to employ freshly graduated dentists to provide preventive measures to children in nurseries and kindergartens in different northern Palestinian districts including fluoride varnish application to all primary teeth as early as possible, educating the teachers as well as the parents about the best hygiene methods and toothpastes to be used by preschool children, making regular dental check-ups to the children, and giving them advices about healthy nutrition.

## Figures and Tables

**Table 1:** Distribution of the population.

Governorate	Male	Female	Total
Jenin	212	146	358
Tulkarem	146	160	306
Nablus	188	143	331
Qabatiya	93	77	170
Toubas	71	57	128
Qalqilya	43	40	83

Total	753	623	1376
Percentage	54.7%	45.3%	100%

**Table 2:** Prevalence of dental caries in primary dentition.

dmft	Number of children	Percentage
Caries-free children (dmft = 0)	330	24%
Children with dmft > 0	1046	76%

Total	1376	100%

**Table 3:** Mothers level of education.

Level of education	Number of mothers	Percentage
Elementary school	305	26.1%
Secondary school	459	39.3%
College graduated	370	31.7%
Postgraduate studies	34	2.9%

Total	1168	100%

**Table 4:** Relationship between mothers' level of education and presence of fillings.

Level of mother's education	Number of children with fillings	Number of children with missing teeth	Number of children with decayed teeth	Number of caries-free children
Elementary school	10	27	286	67
Secondary school	45	43	379	108
College graduated	78	21	257	146
Postgraduate studies	21	7	10	9

Total	154	98	932	330

**Table 5:** Caries prevalence in males and females.

dmft	Males	Females	Total
Caries-free children (dmft = 0)	172	158	330
Children with dmft > 0	581	465	1046

Total	753	623	1376
Percentage	54.7%	45.3%	100%
Caries prevalence	77.2%	74.6%	Average = 76%

**Table 6:** Overall mean dmft scores and scores per component in males and females.

Gender	Decayed (dt)	Missing (mt)	Filled (ft)	dmft
Male				
Mean	2.23	0.16	0.21	2.60
*N*	753	753	753	753
Female				
Mean	1.94	0.07	0.28	2.29
*N*	623	623	623	623

Total				
Mean	2.10	0.12	0.24	2.46
*N*	1376	1376	1376	1376
